# Benchmarking public policies to create healthy food environments compared to best practice: the Healthy Food Environment Policy Index in Guatemala

**DOI:** 10.1186/s13690-022-00928-w

**Published:** 2022-07-19

**Authors:** Carmen María Sánchez-Nóchez, Manuel Ramirez-Zea, Stefanie Vandevijvere, María Fernanda Kroker-Lobos

**Affiliations:** 1 INCAP Research Center for the Prevention of Chronic Diseases, Institute of Nutrition of Central America and Panama (INCAP), Calzada Roosevelt 6-25 zona 11, 01188 Guatemala City, Guatemala; 2 Graduate School, Faculty of Pharmacy and Chemistry Sciences, Universidad de San Carlos de, 11th avenue, zone 12, 01012 Guatemala City, Guatemala; 3 Scientific Institute of Public Health (Sciensano), Unit of Epidemiology, J. Wytsmanstraat 14, B-1050 Brussels, Belgium; 4 Department of Epidemiology and Biostatistics, School of Population Health, Faculty of Medical and Health Sciences, University of Auckland, Auckland, PC. 1010 New Zealand

**Keywords:** Guatemala, Food policies, Healthy food environment, INFORMAS, Food-EPI, Nutrition-related noncommunicable diseases

## Abstract

**Background:**

Benchmarking the implementation of healthy food environment public policies against international best practices may accelerate the government response to prevent obesity and non-communicable diseases (NCDs) in the countries. The aim of the study was to determine the extent of food environment policy implementation in Guatemala and to identify and prioritize actions for the government to accelerate their implementation.

**Methods:**

The INFORMAS Healthy Food Environment Policy Index (Food-EPI from the International Network for Food and Obesity/NCDs Research, Monitoring and Action Support) was used. Evidence of implementation for 50 good practice indicators within the seven food policies and six infrastructure support domains was compiled, and subsequently validated by Guatemalan government officials. A national civil society expert panel on public health and nutrition performed an online assessment of the implementation of healthy food environment policies against best international practices. The level of agreement among evaluators was measured using the Gwet second order agreement coefficient (AC2). The expert panel recommended actions for each indicator during on-site workshops and those actions were prioritized by importance and achievability.

**Results:**

The expert panel rated implementation at zero for 26% of the indicators, very low for 28% of indicators, low for 42%, and medium for 4% of indicators (none were rated high). Indicators at medium implementation were related to the use of evidence for developing policies and ingredient list/nutrition information panels on packaged foods. Seventy-seven actions were recommended prioritizing the top 10 for immediate action. The Gwet AC2 was 0.73 (95% CI 0.67–0.80), indicating a good concordance among experts.

**Conclusions:**

In the Food-EPI of Guatemala, almost all indicators of good practice had a low or less level of implementation. The expert panel proposed 12 priority actions to accelerate policy implementation to tackle obesity and NCDs in the country.

**Supplementary Information:**

The online version contains supplementary material available at 10.1186/s13690-022-00928-w.

## Background

Guatemala experiences the highest prevalence of the double burden of malnutrition in the Western Hemisphere [1]. At national level, one out of two women in reproductive age suffers from overweight and obesity, and one out of two children suffers from stunting. This situation is related to the rapidly increasing prevalence of overweight and obesity in Guatemala during the past few years, particularly in rural and indigenous population groups, which adds to the still high prevalence of stunting [2]. At household level, 28% of indigenous and 14% of non-indigenous households have a mother who suffers from overweight or obesity, and a child under 5 years of age with stunting [2].

“High blood pressure, high fasting blood sugar levels, and overweight/obesity are the top three risk factors for mortality in the Americas” [3]. The excess intake of sugars, fats (total, satured, trans) and sodium is closely linked with these nutrition-related noncommunicable diseases (NCDs: cancers, cardiovascular disease, diabetes and chronic lung illnesses) risk factors [4–8]. Moreover, food environments are defined as “the collective physical, economic, policy and sociocultural surroundings, opportunities and conditions that influence people’s food and beverage choices, and nutritional status” [9]. It has been established that unhealthy food environments are a major driver of unhealthy population diets and obesity [10, 11]. Unhealthy food environments have been previously documented in Guatemala, in particularly high availability and extensive marketing of processed foods high in energy, sugars, saturated fats, and sodium [12–15]. This phenomenon might have contributed to the increase in overweight in Guatemala, as well as in most low- and middle-income countries [1, 2, 10–12, 15]. Government actions are essential to increase the healthiness of food environments and monitoring the degree of implementation of recommended policies is an important part of ensuring progress towards better population nutritional health [16].

**Fig. 1:**
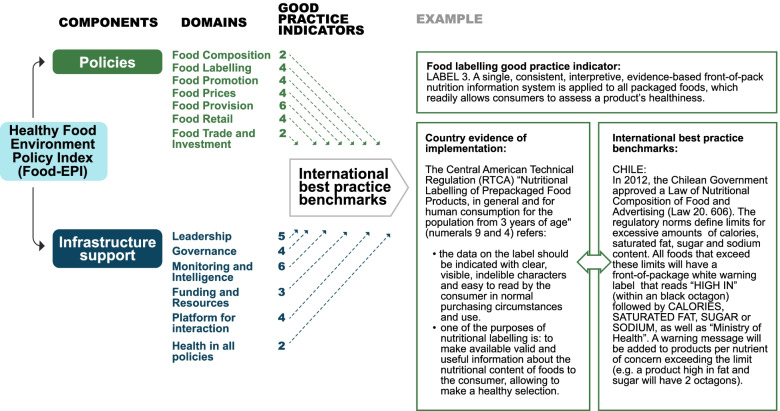
The INFORMAS Food-EPI and benchmarking country evidence of implementation compared to international best practices exemplified [17]. Fig. 1: This figure has been reproduced with permission from INFORMAS

The International Network for Food and Obesity/NCDs, Research, Monitoring and Action Support (INFORMAS) has developed a tool and a process to monitor implementation of public policies on food environments, known as the Healthy Food Environment Policy Index (Food-EPI) (Fig. 1) [16–19]. Through increasing accountability, the Food-EPI has the potential to accelerate policy implementation by governments to reduce obesity and diet-related NCDs. The Food-EPI measures the extent of local implementation of internationally recommended actions and policies compared with international best practices, and formulates concrete actions prioritized both by their importance and achievability [9, 20]. The ratings on the extent of implementation are performed by a civil society expert panel, based on an evidence report document verified by government experts [9]. The Food-EPI provides a useful set of indicators focusing on where government actions are needed most, along with a process that involves a wide range of stakeholders. The Food-EPI has the potential to serve as an educational tool/process, informing participating experts of food environment policies and best practices, and the resulting scorecards and priorities can be used to support advocacy efforts [9, 20, 21]. The roles of evidence in ‘evidence-based policy-making’ are to (i) identify problems; (ii) measure their magnitude and seriousness; (iii) review alternative policy interventions; (iv) assess the likely consequences of particular policy actions, and (v) evaluate the outcomes that result from the policy-making process [22]. The role of advocacy organizations is to identify corporate policies and practices as well as sovereign government policies, and to utilize this information in a strategy to hold different sectors to account for their actions. They will need to press for greater involvement as ‘meaningful stakeholders’ in assessing corporate activities and setting standards for corporate behavior [23]. The Food-EPI indicators are coherent with the list of proposed policy options for Member States included in WHO’s Global Action Plan for the Prevention and Control of NCDs (2013–2020) [24], the WHO’s high level Commission report on ending childhood obesity [25] and the World Cancer Research Fund International NOURISHING framework for Healthy Diets [26, 27]. The Food-EPI was applied in 11 countries between 2015 and 2018. Chile had the largest proportion of policies (13%) rated at “high” implementation, while Guatemala had the largest proportion of policies (83%) rated at “very low if any” implementation. The overall Food-EPI score was “medium” for Australia, England, Chile, and Singapore, while “very low if any” for Guatemala. The policy areas that were most frequently prioritized included taxes on unhealthy foods, restricting unhealthy promotion and front-of-pack labelling. The Food-EPI was found to be a robust tool and process to benchmark governments’ progress to create healthy food environments [28].

In Guatemala, obesity and NCDs have been recognized as public health problems and the Central American Council of Ministries of Health designed a strategy for obesity and NCD prevention. Such strategy includes food environment interventions, such as the implementation of food marketing regulations and front-of-pack labelling. At national level, the National Strategic Plan for the prevention of NCDs 2015–2020 of the Ministry of Health established the need of food labelling regulation, regulation of sodium content and trans fats in processed food, regulation on food advertising, and an excise tax on energy-dense food and beverages with low nutritional quality, among others actions [29]. To date, however, no study has been conducted to evaluate the national response or the extent of implementation of actions to create healthy food environments for the prevention of obesity and NCDs in Guatemala. Consequently, the objectives of the present study were 1) to measure the extent of implementation of public policies on healthy food environments according to the perception of a national civil society expert panel on public health and nutrition using the Food-EPI, and 2) to generate prioritized actions based on the identified implementation gaps, in order to accelerate the implementation of public policies towards healthier food environments.

## Methods

### Establishment of the national expert panel

A cross-sectional study was conducted during 2016 and 2017 with 64 public health and nutrition experts from the civil society of Guatemala (National expert panel). Experts were involved in three different phases: 1) an online questionnaire to assess the extent of implementation of public policies on healthy food environments against international best practices benchmarks; 2) an on-site workshop to recommend and reach consensus on government actions; and 3) a questionnaire to prioritize the actions recommended based on both, importance and achievability.

We consulted the governmental Food and Nutrition Security Secretariat (SESAN), charged with coordinating the National Council for Food and Nutrition Security (CONASAN), to provide a list of institutions related to public health and nutrition from civil society, such as international cooperation agencies, universities, research institutions, and other non-governmental organizations (NGOs). Based on that list, researchers contacted the highest authority from each institution and requested them to assign or delegate an expert from their own institution knowledgeable about governmental policies, plans, programs or projects in: a) nutrition, b) public health, c) food and nutrition security, d) NCDs and/or e) sustainable development. A proper introduction of the components of the Food-EPI and a study registry form were provided to each institution to acquire general information from the appointed professionals, including professional training (academic degrees), as well as general information about the institution. We encouraged participation of experts throughout the country. The potential experts were categorized according to location, gender, and type of organization (universities and research institutions; NGOs such as international cooperation agencies; and other civil society organizations such as Instances of Consultation and Social Participation, professional organizations, national alliances and networks related to food, agriculture and health).

The study protocol was approved by the Institutional Review Board at the Institute of Nutrition of Central America and Panama (IRB # 00007541) and all participants provided a written informed consent.

### Adaptation of instruments

Since this was the first time that the Food-EPI was applied in a Latin-American context, we adapted and translated the Food-EPI tool into Spanish, along with experts from Chile, Mexico, and INFORMAS [30]. The tool was adapted from the original instrument, which has been tested extensively before [20, 21]. Based on the adapted tool, an online questionnaire was developed. This questionnaire was composed by two main components: a) food policies and b) infrastructure support for the prevention of obesity and NCDs. These two components were subdivided into 13 domains and 47 indicators of good practice policies on healthy food environments indicators. Indicators for food promotion in and around schools were specified, and indicators for safe drinking water provision for human consumption were added to the original questionnaire, comprising 50 good practice indicators in Latin America. These indicators were added since food marketing influences preferences and increases children’s requests for food. Child-oriented advertisements are available in almost all stores within a short walking distance from schools, exposing children to an obesogenic environment [15]. Additionally, the NOURISHING framework regarding restricting food marketing has sub-policy areas for regulation of food marketing in schools and regulation of specific marketing techniques for children. This was the reason to split the indicator and its potential specific monitoring process [26, 30, 31]. The new indicators about water provision in schools and public spaces were added in consonance with the world commission on ending childhood obesity recommendations [25]; the plan of action for the prevention of obesity in children and adolescents for the Americas region [31]; and relevant evidence in the countries about impact and barriers for drinking water availability, which continues to be an issue in the majority of Latin America unlike developed countries that do not face the problem [32, 33].

### Compilation of international best practices benchmarks

The INFORMAS framework has compiled a series of policies and regulations regarding international best practices benchmarks and their development has been described elsewhere [9, 20]. In addition to the original best practices benchmarks, we added examples of best practices in food policies from Latin America, such as the front-of-pack warning label system from Chile; food-based dietary guidelines from Brazil; the introduction of a 10% tax on sugar-sweetened beverages in Mexico, among others. The Latin American best practices examples were discussed and agreed with experts from Chile and Mexico.

### Compilation of country evidence on healthy environment public policies

Country evidence related to each of the 13 domains and 50 good practice indicators conforming the Food-EPI was obtained by the research team during the period of July to September 2016 (Fig. 1). The evidence depicted currently active public policies on healthy food environments. To verify information sources, officials from SESAN and member institutions of CONASAN (as Food Security and Nutrition technical coordination institution and multi-sectoral body that leads nutrition policy direction and decision-making at the Government level in Guatemala) were consulted, as well as websites of each of those institutions when needed. We also consulted key government officials by e-mail or in person and asked them to confirm the existence of a policy, norm, or regulation and references to related publications if needed and available. Thirteen documents were generated, one for each component, which included 1) the country evidence, 2) photographs of the original norm or law fragment and 3) references.

### Validation of country evidence

Validation of country evidence was performed by 48 key officials of Ministries, Secretariats, Systems, Councils, Commissions, and Universities related to CONASAN and Food-EPI domains, between October to December 2016. Those key officials had knowledge on policies, plans, programs or projects about: a) nutrition, b) public health, c) food and nutrition security, d) NCDs, and/or e) sustainable development. Participating institutions and number of participants per institution are listed in Table 1. To validate the country evidence, officials were asked by email or in person to register their appreciation about the completeness, accuracy and relevance of the evidence. Additionally, officials were asked to identify and facilitate other documents in case the information was incomplete.

**Table 1:** Participants from Ministries, Secretariats and other governmental institutions who verified the evidence of implementation

Institution	Department/Unit	n
Ministry of Agriculture, Livestock and Food (MAGA)	Vice-Ministry of Food and Nutrition (VISAN) Vice-Ministry of Rural Development (VIDER)	3
Ministry of Communications, Infrastructure and Housing (CIV)	Executive Unit for Road Maintenance (COVIAL)	1
Ministry of Social Development (MIDES)	Monitoring and Evaluation Department	3
Ministry of Economy (MINECO)	Vice-Ministry of Integration and Exterior Trade Directorate of Attention and Assistance to the Consumer (DIACO)	3
Ministry of Education (MINEDUC)	Educational Community Strengthening General Direction Office (DIGEFOCE)	2
Ministry of Finances (MINFIN)	Department of Fiscal Evaluation	2
Ministry of Public Health and Social Assistance (MSPAS)	Non-Communicable Diseases and Cancer Program (PNECNTyC)	1
	Department of Regulation and Food Control (DRCA)	1
	Nutrition Units from the Integrated System of Healthcare -SIAS- in Northeast Guatemala City, El Progreso, Izabal, Jutiapa, Chimaltenango, Sacatepéquez, Quetzaltenango, Totonicapán, Ixcán, north and south Petén.	19
	Education and Health Communication Program (PROEDUSA)	1
Presidential Secretariat of Social Welfare (SBS)	Under-Secretariat for Care and Protection of Children and Adolescents	2
Presidential Secretariat for Planning and Programming (SEGEPLAN)	Department of Institutional and Sectorial Planning. Sector: Social and Public Health	2
Food and Nutrition Security Secretariat (SESAN)	Department of Institutional Strengthening Planning Department	3
National System of Science and Technology (SINCYT)	Centre for Studies of Sensory Impairment, Aging and Metabolism (CESSIAM)^a^	2
National Commission for Non-Communicable Diseases and Cancer	Executive Board	1
National Youth Council (CONJUVE)	Department of Monitoring and Evaluation	1
University of San Carlos of Guatemala	Faculty of Pharmacy and Chemistry Sciences	1
**TOTAL**		**48**

^a^SINCYT designated CESSIAM, which is a Non-Governmental Research Center, dedicated to nutrition and public health research

### Pilot testing of the Food-EPI tool

We used an online platform (REDCap, University of Vanderbilt) to administrate the Food-EPI questionnaire. The online rating process on the level of implementation of healthy food environment policies against international best practices benchmarks was tested in December 2016. Nine of the ten invited experts (different experts from the actual rating process) on nutrition, agronomy and medicine accepted voluntarily to participate in the pilot test and completed the Food-EPI online form. Voluntary experts belonged to the sectors included in the study (five from universities and research institutions, one expert from a non-governmental organization, and three from civil society organizations). On the platform, summaries of the country evidence were presented to the experts as well as the international benchmarks. Based on the results from the pilot test, corrections and adaptations to documents and questionnaires were made.

### Phases of the study

#### Phase 1: rating the healthy food environment policy implementation, using the Food-EPI tool

During the first phase, we sent access data (link and password) to the Food-EPI online questionnaire (REDCap) to the 68 public health and nutrition experts who agreed to participate as part of the national expert panel. Additionally, we shared an introductory video of the study with instructions and contact information in case of questions or doubts.

Experts were given up to 15 days to complete the questionnaire. For each indicator of good practice, experts were asked: 1) to read the country evidence; 2) to compare de country evidence against international best practices benchmarks; and 3) to rate the extent of implementation of public policies in the country against international benchmarks. The extent of implementation was rated based on the following scale: a) less than 20% of implementation compared to best practice, b) between 20 and 40%, c) between 40 and 60%, d) between 60 and 80%, e) between 80 and 100% of implementation compared to best practice. Finally, experts were asked to indicate if they were confident or uncertain when assessing the level of implementation of a given indicator, and to provide comments if necessary.

#### Phase 2: consensus of actions

The second phase consisted of an on-site workshop with members of the national expert panel and it was accomplished during the same month that the Food-EPI questionnaire was completed. Three workshops were carried out in different parts of the country: Guatemala City (Central); Río Hondo, Zacapa (East); and Quetzaltenango, Quetzaltenango (West) to ensure the participation of as many experts as possible. As an introduction to the workshop, a graph showing the scores distribution (obtained from the rating process) and mean scores for each good practice were presented to generate discussion. For each indicator, experts were asked to identify and reach consensus on specific actions for improving the level of policy implementation as a potential route that could be followed by the government.

#### Phase 3: prioritization of actions

During the third phase, members of the national expert panel were invited to prioritize the proposed actions for the government. To determine the importance and achievability of prioritized actions, a Likert scale was created for ranking actions proposed in phase two. For each proposed action, experts were asked to record the level of priority based on the perception of both importance and achievability, according to the following scale and score: very high (5), high (4), medium (3), low (2), very little (1), none (0). Phase 3 took place in Guatemala city (central workshop) and the experts from east and west were online. The tool for east and west experts were sent and received by email.

### Data analysis

The mean scores of the extent of implementation for each indicator, component and domain, were calculated based on the experts’ ratings. The level of implementation was then categorized as follows: high > 75%, medium 51–75%, low 26–50%, and very low < 25%. The inter-rater reliability was calculated with the Gwet AC2 coefficient using AgreeStat software version 2015.5 (www.agreestat.com).

Actions proposed by the civil society were ranked averaging the sum of scores of importance and achievability for each indicator to obtain the level of prioritization. Afterwards, ranks were listed in descending order to identify the first top 10 prioritized actions proposed to the Government of Guatemala.

## Results

### National expert panel participation

A total of 142 organizations from civil society were invited, and 68 public health and nutrition experts accepted to participate in the study. However, 4 experts did not participate in any of the phases, resulting in a final sample of 64 experts who participated in at least one of the study phases (Table 2).

**Table 2:** Participants of the national expert panel who conducted the ratings, proposed actions, and prioritized actions

	Invited*	Agreed to participate**	Phase 1^a^	Phase 2^b^	Phase 3^c^	Experts in all phases	Total of participants***
	(*n* = 142)	(*n* = 68)	(*n* = 45)	(*n* = 55)	(*n* = 39)	(*n* = 29)	(*n* = 64)
**National expert panel**		48	70	86	61	45	94
**By type of organization**							
Universities and research institutions	50	56	58	58	56	59	58
NGOs	23	21	18	20	26	21	19
Other organizations from civil society	27	24	24	22	18	21	23
**By country area**							
West area	8	12	13	16	13	17	14
East area	8	12	9	16	10	7	14
Central area	83	76	78	67	77	76	72
**By gender**							
Female participation	30	68	73	75	82	86	70

*NGOs* Non-Governmental Organizations

All are percentage values

*Estimated number of experts, considering the potential designation of an expert for each invited institution

**Experts who consented to participate in the Food-EPI process

***Final sample of experts who attended to at least one of the phases

^a^Phase 1: Food-EPI online rating

^b^Phase 2: On-site workshop for action recommendations and consensus

^c^Phase 3: On-site/online workshop for prioritizing actions

Fifty-eight percent of the participants were experts from universities and research institutions (academia); 19% from NGOs and 23% from other civil society organizations. Seventy percent of the national experts were female and 98% of all experts had a bachelor’s and/or a master’s degree in nutrition, public health or any other discipline.

### Phase 1: rating the healthy food environment policy implementation, using the Food-EPI tool

**Fig. 2:**
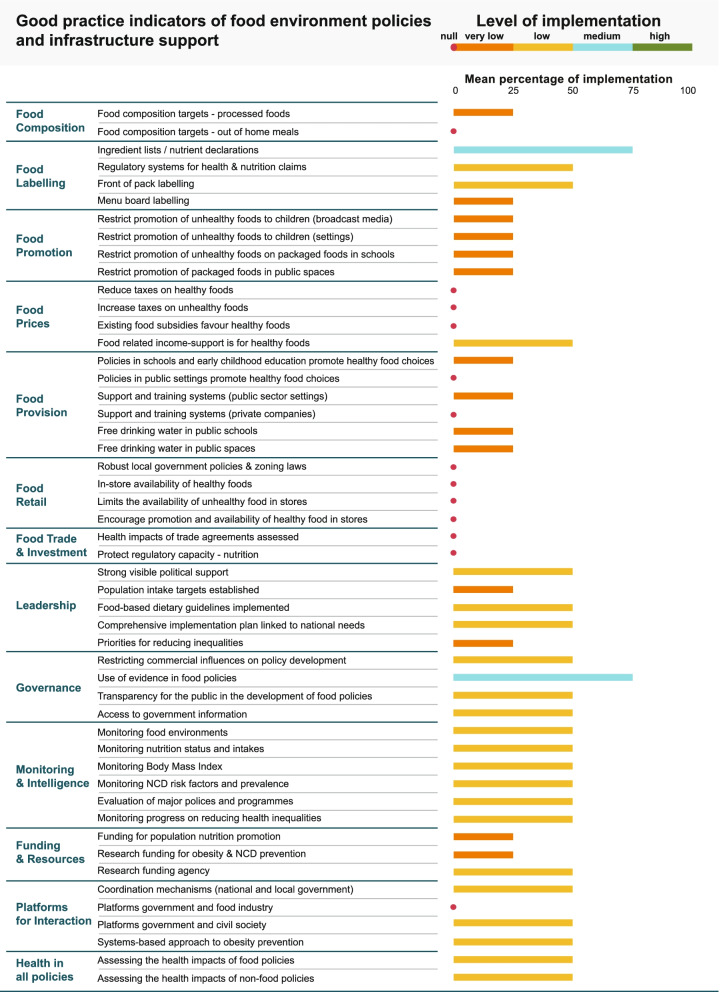
Level of implementation of healthy food environment policies against international best practice by the Government of Guatemala. Fig. 2: Level of implementation (very low < 25%, low 26–50%, medium 51–75%, high > 75%) defined by civil society nutrition and health experts in 2017

In Guatemala, 45 civil society experts rated the level of implementation of each of the 50 good practice indicators compared to international best practices (Fig. 2). Zero implementation was found in 13 indicators of good practices (26%), mostly from food prices, retail and trade, and investment domains. About 14 indicators (28%) of good practice indicators were rated at very low implementation. Twenty-one indicators (42%) resulted in low implementation and only two indicators (4%) reached medium implementation (ingredient list in packaged foods and based-evidence public policy). In general, the infrastructure support component for policy implementation had an overall average score of 31% (low), while the food policies component had an average of 12% (very low). The domains with the highest scores were governance (41%), leadership (31%), monitoring & intelligence (31%), food labelling (31%), and Health in All Policies (30%). The domains with the lowest scores were: food retail (0%), food trade and investment (0%), food prices (9%), food composition (10%), food provision (13%), food promotion (18%), funding and resources (26%), and platform for interaction (27%). The indicators of good practices with the highest scores of implementation are the use of evidence in food policies (54%), ingredient lists and/or nutrient declarations in food labelling (52%), implementation of food-based dietary guidelines (43%), access to government information (43%), transparency in development of food policies (41%), and monitoring nutrition status and intakes (41%).

The inter-rater general reliability during the rating phase was 0.73 (95% CI, 0.67, 0.80), indicating a good agreement among raters. The inter-rater reliability was higher among researchers (0.83; 95% IC 0.78, 0.89), followed by university academics (0.77; 95% IC 0.71, 0.84), NGOs experts (0.68; 95% IC 0.59, 0.79), and other organisms from civil society (0.65; 95% IC; 0.56, 0.74).

### Phases 2 and 3: consensus and prioritization of actions

Fifty-five experts distributed across the three regions recommended a total of 77 different actions on the 50 indicators to the government. Then, during the same month, 39 experts participated in the third phase, in which they prioritized seven food policies and five infrastructure support actions in terms of importance and achievability (Table 3).

**Table 3:** Top 10 priority actions, recommended by civil society, to accelerate progress towards prevention of NCD’s

Domain	Prioritized Action^**a**^	Rank
** Food Policy Actions**		
Food Labelling	To establish within the Central American Technical Regulation -RTCA-, sugar and added sugars declarations as well as a new format for ingredients lists and nutrients declarations (size of legend, position of translated information, among others).	3
Food Provision	To guarantee and monitor the provision of safe drinking water, free-of-charge, in all schools.	4
Food Labelling	To define based-evidence standards for monitoring health and nutrition claims, to avoid misleading claims on food packages.	5
Food Provision	To support the healthy schools’ initiative, as well as provision of fruits and vegetables at the school-feeding program, creating a system to purchase products directly from cooperatives and local farmers.	7
Food Labelling	To assess the nutritional content of packaged food products during the process of sanitary registration, to verify the use of permitted nutritional claims and to avoid misleading claims.	8
Food Prices	To create based-evidence nutritional standards (defined by experts from civil society), to assess the nutritional quality of foods offered in social programs, especially in the school-feeding program, without any influence from the food industry to avoid conflict of interest.	10a
Food Provision	To guarantee and monitor the provision of safe drinking water, free of charge, in all public places.	10b
** Infrastructure Support Actions**		
Governance	To use the best scientific evidence available on the contribution of food environments on population diets and update the National Food and Nutritional Security Policy.	1
Governance	To disseminate evidence on nutrition and NCDs for designing public policies and guiding the implementation of actions.	2
Leadership	To create an integrated social policy for sustainable human development, with the participation of the public sector and civil society to: a) Establish, as a priority, overweight, obesity and NCD’s’ prevention in children as part of the agenda of the President and Ministries and b) Strengthen the infrastructure support in the country with the existing platforms at national, departmental and municipal levels.	6
Leadership	To establish a base-evidence action plan (taking into account the WHO’s Plan) with attainable and measurable targets, focused on the reduction of critical nutrients (fat, sugar, sodium, calories) in all processed foods.	9a
Leadership	To place and prioritized a public agenda in the Ministry of Health, the National Plan for the prevention of NCDs and the National Commission for the prevention of NCDs.	9b

^a^77 actions were recommended and agreed by the experts. The “top ten” actions are 12 recommendations. Actions sharing same ranks are identified with letters

“*To guarantee and monitor provision of safe drinking water, free of charge, in all schools*” was the most important action prioritized by experts. The most achievable action according to the experts was *“to disseminate evidence on nutrition and NCDs for designing public policies and guiding the implementation of actions*”. The most important and achievable corresponded to the governance domain *“to use the best scientific evidence available on the contribution of food environments on population diets and update the National Food and Nutritional Security Policy”.*

As a final step, researchers also identified prioritized actions within each component by sorting scores from high to low (Supplementary Table 1S). This ranking allows for the visibility of some actions with high importance but considered as non-feasible by the experts. Some actions raised by the experts that did not reach the top 10, but with high ranking within the policy domain, are: to foster research on nutrient profiling of food products; regulations on food advertising targeted to children; regulation of critical nutrients in packaged products; and to guarantee the supply of fruits and vegetables in local municipalities. Overall, experts emphasized that food policy and infrastructure support actions need to be encouraged at municipal level to accelerate the implementation of actions aimed at improving the local food environments.

## Discussion

According to the Food-EPI tool, 96% of the good practice indicators had none, very low or low level of implementation, compared with international best practice benchmarks. The experts recommended and prioritized 12 actions to create a healthier food environment, representing a potential healthy food policy package that could be implemented by the -Government of Guatemala.

Scores in Guatemala are lower than the ones found in Mexico, where experts rated several indicators at medium (51–75%) and none at zero implementation. Comparatively, the Mexican indicators rated lower were, evaluation and monitoring of the food retail policies (10%); restricting the density of fast-food restaurants and convenience stores (9.2%); and incentives to increase the availability of healthy foods in stores (7.2%); whereas the lower indicator in Guatemala was menu board labelling (10%), additionally to the other 13 indicators rated as null implementation. Furthermore, regarding the Latinamerican best practices, the Mexican tax applied to unhealthy food and beverages indicator was included as a benchmark because of its pioneer regulation on sugar-sweetened beverages [30]. In contrast, scores in New Zealand showed that 46% of indicators were rated as medium and high in terms of implementation [21].

In comparison with other 10 countries where Food-EPI has been measured, Guatemala scored the lowest in the implementation of food policies. However, the overall score on the infrastructure support indicators were similar to those found in more developed countries, suggesting that in Guatemala, infrastructure support might not be the main obstacle to implement healthier food environment policies [28].

The low implementation of policies on healthy food environments in Guatemala might be explained by the fact that the National Policy of Food and Nutrition Security does not prioritize populations suffering from overweight or obesity as a vulnerable group [34, 35]. In contrast, the most achievable action identified by the experts to support a healthy food environment is to disseminate evidence on nutrition and NCDs for designing public policies and guiding the implementation of actions. The most prioritized action is to use the best scientific evidence available on the contribution of food environments on population diets and update the National Food and Nutritional Security Policy. In addition, policies and monitoring to protect the population from consuming foods with excessive amounts of fat, sodium, energy and sugar have not been a priority for the government, combined with a weak empowerment from civil society to demand regulatory policies and accountability systems. The food labelling indicator reached a medium level of implementation, since the Central American Technical regulation follows the CODEX voluntary guidelines, which includes ingredient list and nutritional declarations on food packages [36]. However, an effective and evidence-based front-of-pack labelling system is absent in the country [37, 38].

Platforms for interaction, leadership, and funding and resources domains obtained none to very low implementation, which is in line with the deficient implementation of food policies for preventing obesity and NCDs. According to the experts, this perception is related with poor allocation of resources by the government to prioritize research and reduction of inequalities associated with obesity and NCDs.

The use of evidence in policymaking obtained a medium level of implementation, since the Strategic Plan for Food and Nutrition Security 2016–2019 emphasizes the relevance and impact of early life nutrition, from conception to 2 years of age on human capital [39]. However, government policies and programs do not yet recognize consistently double-duty actions for nutrition, that include the prevention of overweight and obesity over the life course and have the potential to improve nutrition outcomes across the spectrum of malnutrition, through integrated initiatives, policies, and programs [40].

The most important action recommended by the experts to guarantee a healthy food environment is the provision of safe drinking water, especially in all schools. Mandatory declarations of sugars and added sugars, changes in the food labelling format, and regulatory systems for health and nutrition claims were other actions prioritized by the experts. Restricting unhealthy food advertising to children was considered important but did not reach the top 10 actions due to feasibility concerns.

Some actions, such as discouraging consumption of sugary drinks by increasing the price through an excise tax, were not found among the prioritized actions despite being described as an international best practice for Latin America [41]. The forgoing is likely because experts opt for actions at the municipal level, instead of those at national level that may require more time and resources for implementation. Remarkably, all the actions identified by the experts were contemplated in the National NCDs plan 2015–2020, and this connection is relevant for the government [29].

The strengths of the study include the focus on the use of a validated methodology from the INFORMAS framework, which has been adapted to Latin America and consequently allowing its comparison across the region and other countries worldwide. Another strength is the broad participation of universities, research institutes, NGOs, and other organizations from civil society from different regions and departments. In the present study, participation of the expected panel of experts and the acceptable agreement score (very good or good) among the experts are similar as those found other countries [28]. Additionally, the country evidence validation process provided a unique opportunity to disseminate international healthy food environment benchmarks to institutions from the public sector. This outreach was a relevant experience for several officers from different Ministries and Secretariats, which might encourage the implementation of actions that could generate healthy food environments in the country. In addition, the individual online assessment process was adjusted to their time availability, so the risk of potential biases that may have occurred during a collective assessment was minimized. Additionally, we used a methodology that could be periodically repeated before the end of each new government, in order to compare progress within the country.

Although the indicators have been extracted from existing overarching high-level policy documents, one limitation is the insufficient or weak international examples that some indicators from the infrastructure support component have. Another limitation is the number of indicators, which makes rating and recommendation of actions time consuming. Regarding the methodology to reach consensus on proposed actions, a prior mechanism of prioritization of indicators could optimize the time used for discussion and agreement. This may, in turn, result in top actions without having to perform a prioritization process afterwards.

As discussed, the Food-EPI has the potential to accelerate policy implementation by the governments to reduce obesity and diet-related NCDs through increasing accountability. Public health and nutrition experts from civil society recommended 12 actions for the multisectoral promotion of healthy food environments emphasizing the urgency of acting at municipal level. Additionally, the Food-EPI represents a prospective healthy food policy package that could be implemented by the government of Guatemala. In 2018, the Parliamentary Front against Hunger presented the “Healthy Eating Promotion” bill to the congress in Guatemala [42]. The bill comprises several of the Food-EPI-recommended actions by the experts, such as: 1) implementation of evidence-based front-of-pack nutritional warnings labeling system; 2) regulations on health and nutrition claims; and 3) regulation of the food marketing targeted to children [42]. If approved, it would be the first country initiative to accelerate progress towards reaching healthier food environments and consequently towards preventing obesity and NCDs in the country. The Food-EPI in Guatemala represents a baseline benchmark for future policies, especially if the aforementioned bill is approved. Additionally, current findings could catalyze the progress of the “Healthy Eating Promotion” congressional bill as well as the efforts of the National Commission of NCDs.

## Conclusion

In conclusion, the Food-EPI in Guatemala showed that almost all good practice indicators had low or null level of implementation. Experts recommended 77 actions, including 12 priority actions through a multisectoral approach for the promotion of healthy food environments for preventing obesity and NCDs. Emphasis was placed on: the contribution of food environments to improve population diets for the update of the National Food and Nutritional Security Policy; evidence dissemination on nutrition and NCDs for public policies and actions; the establishment of sugar and added sugars declarations as well as new formats for ingredient lists and nutrients declarations in food labelling; safe water provision in schools; and the urgency of acting at municipal level on healthy food environment policies and infrastructure support to prevent obesity and NCD´s.

## Supplementary Information

**Additional file 1: Supplementary Table S1.** Priority actions for the Guatemalan government, within each Food-EPI domain, recommended by civil society.

## Data Availability

Data contain potentially identifying information. Due to privacy considerations imposed by the IRB of the Institute of Nutrition of Central America and Panama (INCAP), the data are not publicly available, however data could be available upon request. Requests for access to the data may be made to the INCAP’s IRB and corresponding author by researchers whose activities are reviewed by a Research Ethics Committee and who agree to sign an appropriate confidentiality agreement. President of INCAP’s IRB: Valentina Santacruz vsantacruz@incap.int.

## Abbreviations

CESSIAM: Centre for Studies of Sensory Impairment, Aging and Metabolism
CIV: Ministry of Communications, Infrastructure and Housing
CONASAN: National Council for Food and Nutrition Security
CONJUVE: National Youth Council
COVIAL: Executive Unit for Road Maintenance
DIACO: Directorate of Attention and Assistance to the Consumer
DIGEFOCE: Educational Community Strengthening General Direction Office
DRCA: Department of Regulation and Food Control
Food-EPI: Healthy Food Environment Policy Index
INFORMAS: International Network for Food and Obesity/NCDs Research, Monitoring and Action Support
MAGA: Ministry of Agriculture, Livestock and Food
MIDES: Ministry of Social Development
MINECO: Ministry of Economy
MINEDUC: Ministry of Education
MINFIN: Ministry of Finances
MSPAS: Ministry of Public Health and Social Assistance
NCDs: Non-Communicable Diseases
NGOs: Non-Governmental Organizations
ODAN: Right to Food Observatory
PNECNTyC: Non-Communicable Diseases and Cancer Program
PROEDUSA: Education and Health Communication Program
RTCA: Central American Technical Regulation
SBS: Presidential Secretariat of Social Welfare
SEGEPLAN: Presidential Secretariat for Planning and Programming
SESAN: Food and Nutrition Security Secretariat
SINCYT: National System of Science and Technology
VIDER: Vice-Ministry of Rural Development
VISAN: Vice-Ministry of Food and Nutrition
WHO: World Health Organization
