# Supplementary material for: Benchmarking public policies to create healthy food environments compared to best practice: the Healthy Food Environment Policy Index in Guatemala
Source: Arch Public Health. 2022 Jul 19;80:174. doi: 10.1186/s13690-022-00928-w (PMC9295498; doi:10.1186/s13690-022-00928-w)
Supplement: Supplementary file 1 — Additional file 1: Supplementary Table S1. Priority actions for the Guatemalan government, within each Food-EPI domain, recommended by civil society. [file 13690_2022_928_MOESM1_ESM.docx]

**Additional file 1**

**Supplementary material S1**

Priority actions for the Guatemalan government, within each Food-EPI domain, recommended by civil society.

| **Domain** | **Prioritized action by domain*** | **Rank**** |  |
| --- | --- | --- | --- |

**Policy actions**

| Food composition | To regulate the content of critical nutrients through a stepwise reduction in packaged foods (including food additives) through establishment of evidence-based cut-offs defined by civil society experts for permitted limits. | 17a |
| --- | --- | --- |
| Food  labelling | To establish within the Central American Technical Regulation -RTCA-, sugar and added sugars declarations as well as a new format for ingredients lists and nutrients declarations (size of legend, position of translated information, among others). | 3 |
| Food  promotion | To restrict unhealthy food marketing targeted to children and adolescents in all massive media (TV and radio). “Unhealthy” defined by evidence-based cut-offs for each critical nutrient. | 16 |
| Food  prices | To create evidence-based nutritional standards (defined by experts from civil society), to assess the nutritional quality of foods offered in social programs, especially in the school-feeding program, without any influence from the food industry to avoid conflict of interest. | 10a |
| Food provision | To guarantee and monitor the provision of safe drinking water, free-of-charge, in all schools. | 4 |
| Food  retail | To guarantee the supply of fruits and vegetables at municipal level along with educational campaigns to highlight the distinctive value based on local regions. | 20 |
| Food trade & investment | To promote research for creating nutritional standards, as well as monitoring and evaluating the potential impacts on population health of imported and exported packaged products containing excessive amount of critical nutrients and food supplements. | 14 |
|  | **Infrastructure support actions** |  |
| Leadership | To create an integrated social policy for sustainable human development, with the participation of the public sector and civil society to: a) establish overweight, obesity and NCD prevention in children as a priority, as part of the agenda of the President and Ministries; and b) strengthen the infrastructure support in the country with the existing platforms at national, departmental and municipal levels. | 6 |
| Governance | To use the best scientific evidence available on the contribution of food environments on population diets and update the National Food and Nutritional Security Policy. | 1 |
| Monitoring & intelligence | To monitor trends on a) consumption of foods with excessive content of critical nutrients, and b) nutritional status of the population through an integrated NCDs surveillance system, including existing national surveys and related systems. | 12c |
|  | To allocate specific funding for monitoring and evaluation of the design of public policies and programs. | 12b |
| Funding & resources | To strengthen the leading role of the Ministry of Health for allocating resources for population nutrition and prevention, as opposed to the current curative medical care. | 24d |
| Platforms for interaction | At municipal level, to strengthen the System of Development Councils and Commissions of SAN for implementing interventions towards healthier food environments and NCD prevention. | 25b |
|  | To institutionalize the “Right to Food” Observatory (ODAN), with emphasis on monitoring healthy food environment policies. | 25c |
| Health in All Policies | To update the current National Policy of Food and Nutrition Safety, including all aspects related to healthy food environments, double burden of malnutrition and NCD prevention. | 12d |

SAN, Food and Nutrition Safety (acronym in Spanish).

*Refers to the action with the highest score within that domain.

**The maximum rank was 48. Actions sharing same ranks are identified with letters.
